# Developmental Cellular Programming: stage-specific architecting muscle and adipose tissues for high-quality meat production

**DOI:** 10.1186/s40104-026-01475-y

**Published:** 2026-08-19

**Authors:** Bo Wang

**Affiliations:** State Key Laboratory of Animal Nutrition and Feeding, College of Animal Science and Technology, China Agricultural University, Beijing, 100193 PR China

**Keywords:** Developmental programming, Intramuscular fat, Meat quality, Muscle

## Abstract

The rising global demand for animal protein has driven intensive selection for rapid growth and leanness, often at the expense of meat quality and animal health. Achieving high-quality meat production with optimal intramuscular fat (IMF) and superior myofiber characteristics remains a major challenge in animal agriculture. Here, we propose a holistic conceptual framework termed Developmental Cellular Programming (DCP), which utilizes distinct developmental windows to precisely regulate skeletal muscle and adipose tissue formation in meat animals. Unlike traditional late-stage finishing strategies, the DCP framework operates as a coordinated, stage-specific pipeline: optimizing maternal nutrition during gestation to maximize prenatal myofiber hyperplasia and initiate adipocyte lineages; executing targeted neonatal interventions to drive microenvironment-mediated adipogenic precursor expansion and angiogenesis; and fine-tuning nutrient allocation during the finishing phase to control cellular hypertrophy and lipid deposition. Within this framework, vascularization is established as a critical, shared control point that coordinates multi-tissue niches to synchronize myofiber and adipocyte development. While advanced three-dimensional cell culture models, such as vascularized tissue organoids, have emerged as powerful platforms for high-throughput screening of key candidate metabolic modulators, bridging the gap to livestock production necessitates translating these cellular responses into whole-animal phenotypes. We propose that future research should focus on conducting in vivo animal trials to elucidate how various nutrients regulate muscle and adipose cell differentiation at specific developmental stages, thereby establishing precision nutritional strategies based on the DCP theory for high-quality meat production.

## Introduction

The global demand for animal protein has spurred decades of intense genetic selection and refined nutritional strategies, leading to remarkable gains in livestock production efficiency. In broiler chickens, for instance, growth rates increased by 400% and feed conversion ratios (FCR) decreased by 50% between 1957 and 2005 [1]. A key driver of this efficiency is the preferential accretion of lean tissue over fat, as muscle growth is far more metabolically efficient [2]. Consequently, modern meat animals like broilers, pigs, and beef cattle have been selectively bred for dramatically leaner body compositions [3].

Historically, this progress has been guided by a narrow focus on macroscopic, whole-body metrics. Selection has prioritized traits like total lean mass growth, largely ignoring the underlying cellular architecture, specifically, the number and size of muscle fibers and adipocytes. This oversight of fundamental prenatal and neonatal developmental processes has come at a cost. The push for extreme muscularity and leanness is now linked to a high incidence of meat quality defects, such as white striping, wooden breast, and pale, soft, and exudative (PSE) meat [4, 5]. These abnormalities are often rooted in excessive muscle fiber hypertrophy, a shift towards glycolytic fiber types, and reduced vascular density [6, 7]. Furthermore, the resulting leaner meat is frequently criticized by consumers for being tough and lacking the flavor associated with intramuscular fat (IMF) [8, 9]. In response, efforts to enhance marbling and fatness through breeding and dietary fattening have revealed significant trade-offs. Breeds with a high genetic potential for IMF, such as Chinese indigenous pigs or Iberian pigs, often deposit excessive subcutaneous and visceral fat, which is less edible and costly to produce [10–12]. Similarly, high-energy diets can boost IMF but simultaneously increase overall body fat mass, reducing meat yield [13, 14], and potentially triggering metabolic dysfunctions that compromise animal health and welfare [15, 16].

These recurring limitations highlight a fundamental biological constraint. Meat is a cellular product, the composition of which is determined by the number and size of muscle fibers and adipocytes. For major domestic species like chickens, pigs, and cattle, the majority of these cells are established prenatally or in the neonatal period [17–21]. Consequently, current production strategies, which are applied postnatally, are operating upon a cellular framework that is already largely fixed. Therefore, the ultimate determinants of growth potential and meat quality, which are the control of myocyte and adipocyte hyperplasia and the resulting cellular composition, are established before conventional breeding and nutritional interventions can take full effect.

In this review, we propose a stage-specific developmental programming approach for optimizing skeletal muscle and intramuscular fat composition. We discuss the developmental biology underlying the formation of muscle fibers and adipocytes, identifying the critical prenatal and neonatal periods that define their ultimate number and size. We also evaluate evidence for targeted interventions, particularly through maternal nutrition and genetic selection, that can modulate hyperplasia and hypertrophy within these developmental windows. By integrating findings across muscle and adipose biology and species lines, we present the Developmental Cellular Programming (DCP) framework for reconciling growth efficiency with superior meat quality and animal welfare. This approach posits that sustainable, high-quality meat production depends not on postnatal regulation, but on the precise, early-life programming of muscle and adipose cellular architecture.

## The hypertrophic limit and meat quality defects

The development and mass accumulation of livestock skeletal muscle and adipose tissues are fundamentally driven by two distinct cellular processes: hyperplasia, which is an increase in cell number, and hypertrophy, which is an increase in cell size. For muscle tissue, prenatal development is primarily driven by hyperplasia to establish the total myofiber number, which largely ceases near or shortly after birth, meaning that postnatal muscle growth relies almost exclusively on myofiber hypertrophy via satellite cell fusion and protein deposition. In contrast, adipose tissue retains the capacity for both hyperplasia and hypertrophy postnatally, where adipose hyperplasia involves the recruitment and differentiation of preadipocytes into adipocytes, to define the lipid storage capacity, and adipose hypertrophy represents the subsequent triglyceride accumulation within adipocytes. Crucially, while both mechanisms contribute to balanced tissue growth, the industrial over-reliance on late-stage cellular hypertrophy to maximize carcass yield often pushes cells past their physiological thresholds. It is this excessive hypertrophy of both myofibers and adipocytes rather than controlled hyperplasia that disrupts cellular homeostasis, induces metabolic stress, and triggers tissue hypoxia, serving as the fundamental root cause of subsequent poor meat quality and compromised animal health.

### The growth-quality trade-off in the livestock industry

Historically, the primary goal of the meat livestock industry has been efficiency—producing the most meat at the lowest feed cost. Modern commercial broilers exemplify this pursuit, reaching growth rates exceeding 60 g/d and achieving slaughter weight in less than six weeks [22]. Such intense growth results in muscle fibers expanding more than fivefold [23, 24]. However, because the number of muscle fibers does not increase after hatching [25], this post-hatch growth is accomplished solely through the enlargement of existing fibers—a process dependent on satellite cells (SCs) fusing into muscle fibers [26, 27].

Compared to slow-growing broilers, fast-growing birds develop larger muscle fibers [28] and a higher proportion of glycolytic fibers [29, 30], a profile associated with limited capillary networks and increased muscle degeneration [28]. As muscle fibers enlarge, vascular density declines [31], leading to inadequate capillary blood supply, hypoxia, and ultimately tissue damage and myopathies such as white striping (WS) and wooden breast (WB) [6, 32–37]. This muscular shift toward glycolytic fibers, which grow more rapidly and require less oxygen than oxidative fibers, represents an adaptive response to genetic selection for high growth rates [38].

Muscle repair depends on SCs. In fast-growing animals, however, this essential cell pool is diminished. For example, in commercial broilers, SC activity peaks shortly after hatching [27], but their abundance declines sharply from over 30% to below 10% within the first week post-hatch and remains at only about 1% by 42 days of age [34]. Similarly, in pigs, the highly selected Landrace breed possesses only half the SCs found in the native Chinese Lantang pig [39]. This depletion results from two primary factors: high growth rates consume SCs rapidly, while limited capillary space impairs SC self-renewal. Angiogenesis and myogenesis are spatiotemporally linked, with SCs engaging in reciprocal interactions with endothelial cells to support muscle regeneration [40]. SCs stimulate angiogenesis [41] and recruit capillaries to form a juxtavascular niche that promotes SC maintenance and renewal [42]. Thus, vascular density critically influences SC proliferation capacity [43]. Greater vascularity increases SC numbers and helps prevent muscle dystrophy [42, 44]. Notably, WS and WB muscles are marked by blood vessel inflammation [45, 46] and reduced vessel density [47], compromising the vascular support needed for effective SC-mediated repair [40, 41, 48]. Therefore, both functional SCs and adequate vascularization are essential to counteract myopathy development.

Comparisons between wild and domestic pigs further highlight these relationships. Wild pigs exhibit twice the capillary density of domestic pigs, along with muscle fibers approximately 25% smaller in cross-sectional area [49]. In pigs with larger muscle fibers, each capillary supplies a greater myofiber area, driving a shift toward fast-twitch glycolytic fibers in domestic breeds [50]. Selection for rapid growth thus promotes greater muscle mass but also leads to lower relative heart weight, poorer vascularization, and a higher incidence of wooden breast myopathy [51]. A high proportion of glycolytic fibers is also linked to pale, soft, exudative (PSE) meat [52]. In contrast, comparing to commercial breeds, local pig breeds maintain higher capillary density and a greater share of oxidative fibers, supporting more efficient aerobic metabolism and contributing to their recognized flavor traits and enhanced fat deposition [11].

### Muscle fiber hyperplasia vs. hypertrophy: divergent effects on health and quality

Cell size and number are fundamental determinants of cellular function, muscle health and meat quality. At the cellular level, size is intrinsically linked to efficiency [53]. The surface-area-to-volume ratio decreases as a cell grows, leading to longer diffusion distances for oxygen and nutrients [53–55]. This inefficiency is compounded by a reduced number of nuclei per tissue volume, which increases intracellular transport distances for molecules like mRNA and proteins [56]. To compensate, larger cells may require more energy for active transport to maintain vital functions, as seen with the Na^+^/K^+^ pump [57]. In contrast, smaller cells with higher surface-area-to-volume ratios are more metabolically efficient, which is crucial for high-energy demands. For example, flight muscles, which have high metabolic demands, are composed of smaller cells to facilitate rapid oxygen uptake [58]. While muscle cells can adapt by increasing and distributing nuclei to support larger sizes [59], researchers have proposed that cell size regulation is a fundamental guardian of cellular fitness and metabolic activity [53].

Excessive muscle fiber hypertrophy is associated with myopathies. Large, heavy-type chickens, for example, have reduced interstitial space between muscle fibers, which limits the capillary networks needed for oxygenated blood supply [31, 60]. The compromised blood supply, hypoxia, and oxidative damage that occur during muscle hypertrophy are major causes of muscle degeneration [32]. Meanwhile, the continuous enlargement of muscle fibers leads to satellite cell exhaustion, which can impair the process of muscle regeneration [61, 62]. In high growth chicken, failure of muscle regeneration in the limitation of satellite cells and blood supply leads to white striping or wooden meat, which are two types of muscle abnormalities commonly seen in broiler production [6, 34, 63].

**Fig. 1:**
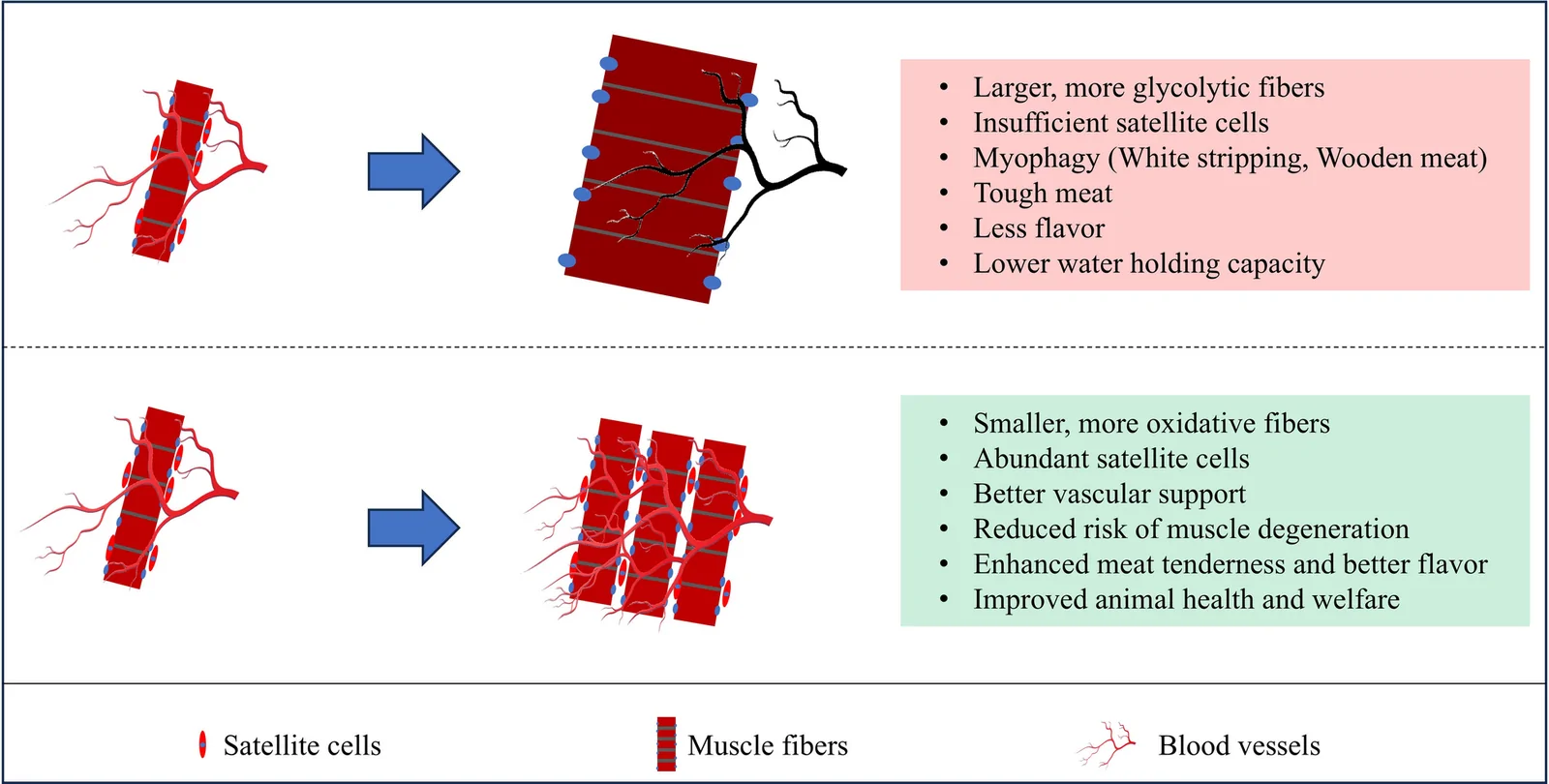
The impact of muscle hyperplasia and hypertrophy on livestock health and meat quality

The size and number of muscle cells also have a profound effect on the quality of meat, including tenderness, water-holding capacity, and flavor. Larger fibers are generally associated with tougher meat, as indicated by a positive correlation between muscle fiber diameter and the shear force required to cut uncooked meat [64, 65]. The diameter of glycolytic muscle fibers tends to increase more rapidly than that of oxidative muscle fibers [38]. Thus, pigs with larger carcass weight have a higher ratio of glycolytic (type IIB) fibers over oxidative (type I and IIA) fibers, which can negatively affect tenderness [66]. Fast-twitch glycolytic (type IIB) fibers have lower phospholipid content, which can diminish the meat's flavor [67]. A rapid postmortem pH drop in muscles with a high proportion of type IIB fibers can cause myofibril contraction, leading to increased drip loss, decreased tenderness, and less flavor [68]. Figure 1 summarizes the impact of muscle hyperplasia and hypertrophy on livestock health and meat quality.

Double-muscling provides an example of enhanced muscle hyperplasia and hypertrophy. In these animals, permanent loss of myostatin function dramatically increases prenatal muscle fiber number—a manipulation that, in principle, aligns with the DCP goal of enhancing hyperplasia [69]. Yet despite this favorable starting cellular architecture, double-muscled cattle and pigs still exhibit pronounced postnatal fiber hypertrophy. MSTN mutation greatly improved meat production in livestock, but the animals are not stronger with higher muscle mass. On the contrary, MSTN mutation poses significant health challenges, including susceptibility to stress, respiratory diseases, and calving difficulties because the excessive muscle mass is a heavy burden for the whole body [70]. In terms of meat quality, the impact of MSTN mutation is complex and multifaceted. In Japanese quail [71, 72], pig [73] and cattle [74], myostatin mutation leads to more fast-twitch glycolytic muscle fibers in large size and increases cooking loss [71, 72]. Interestingly, despite this shift in fiber type, meat from MSTN-mutated animals can exhibit improved tenderness in some cases. For instance, MSTN-mutated pork is more tender due to a significant reduction in collagen content [75]. Similarly, while double-muscled (DM) Belgian Blue cattle possess a higher proportion of type IIB and IIA fibers compared to breeds like German Angus [74], many studies report that DM beef is more tender, primarily attributed to its lower collagen content [70, 76, 77]. DM cattle meat have a lower background toughness largely because of its lower collagen content [78]. However, these tenderizing effects are not consistent and are frequently counterbalanced by other quality defects. Some studies found no tenderness improvement in DM beef [79], and it can even be tougher than conventional beef after aging, due to reduced activity of key proteolytic enzymes (calpain, calpastatin, and cathepsin) [78]. Furthermore, DM beef typically exhibits higher drip and cooking losses [78]. Coupled with an extremely low intramuscular fat content [78], these traits result in meat that is often perceived as less juicy and flavorful [70, 79]. Thus, increasing muscle fiber number without concurrent control of postnatal muscle hypetrophy will still lead to quality defects.

### Adipocyte hyperplasia and hypertrophy and their impact on meat quality

Like muscle, fat tissue grows by adipocyte hyperplasia and hypertrophy. In livestock, adipocyte hypertrophy generally happens during fattening. Excessive energy is converted into triglycerides and stored in adipocytes as lipid droplet, which leads to enlargement of individual cells. Adipocyte hypertrophy is a primary driver of metabolic dysfunction in animals. While adipocytes show high plasticity, enlargement of the cells increases the tension and stiffness of the extracellular matrix (ECM), leading to fibrosis, inflammation, and metabolic dysfunction like insulin resistance, as the tissue struggles to expand, causing excessive deposition and alignment of collagen and other ECM proteins [80, 81]. The dysfunctional hypertrophic adipocytes secrete pro-inflammatory adipokines such as tumor necrosis factor-α (TNF-α) and interleukin-6 (IL-6) [82], which promote macrophage infiltration and the formation of crown-like structures within the fat pad [83, 84]. This chronic low-grade inflammation within adipose tissue is a key contributor to systemic insulin resistance and the ectopic deposition of lipids in the liver and muscle [85]. Obesity-related metabolic dysfunction is common and causes significant economic burden in dairy cow [86]. However, this is rarely observed or has been ignored in livestock raised for meat because they are generally slaughtered at a very young age. However, excessive fat deposition in livestock leads to lower lean muscle yields, an increased need for carcass processing and trimming of fat tissues [87].

**Fig. 2:**
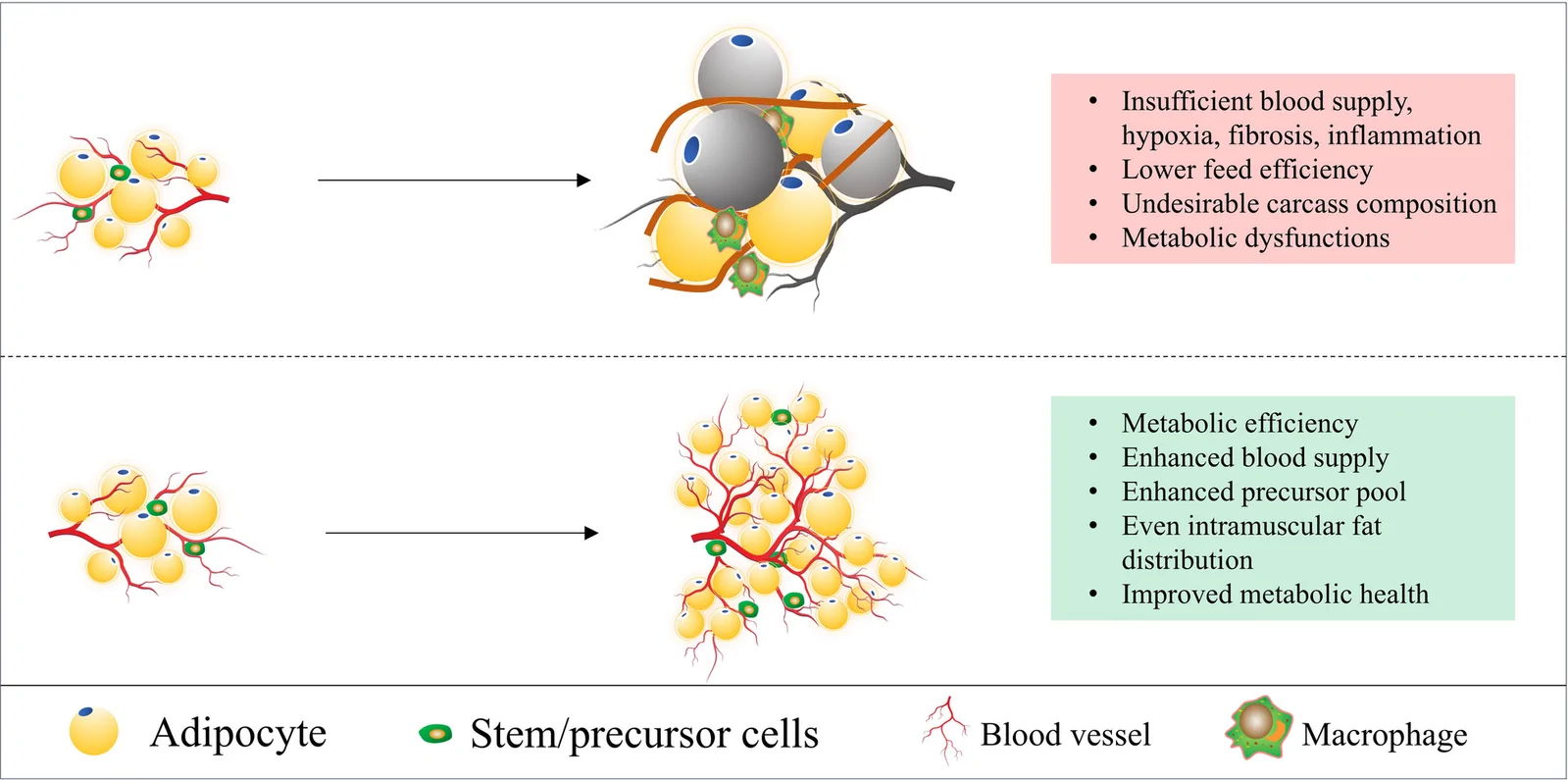
The impact of adipocyte hyperplasia and hypertrophy on livestock health and meat quality

Adipocyte hyperplasia, which is the process of generating new, small adipocytes from progenitor cells, facilitates a healthy expansion of adipose tissue. This increase in cell number enhances the tissue’s overall energy storage capacity without overburdening individual adipocytes [88, 89]. Individuals with a greater capacity for hyperplastic expansion maintain better glucose homeostasis despite elevated fat mass, primarily due to their smaller adipocyte size [90]. In sheep, breeds with a hyperplastic tail fat depot are better equipped to manage energy fluctuations in harsh environments [91]. Figure 2 shows the impact of adipocyte hyperplasia and hypertrophy on livestock health and meat quality.

Fat has its own unique taste, called oleogustus [92], which is primary provided by fatty acid [93]. Other structural components of adipocytes and ECM would contribute to the textural cues (mouthfeel) of fat. Adipocyte size and number change the lipid content in a piece of subcutaneous or visceral fat but how would this affect meat quality is unknown, nevertheless, those fats are rarely eaten directly in most regions. For intramuscular fat, cell number plays a greater role in fat deposition than cell size [94]. De novo adipogenesis increases the sites for fat storage which potentially contributes to well distribution of intramuscular fat. Adipocyte hypertrophy increases the size of marbling flecks but is less desirable for consumers.

### Methodology for cell number and size quantification

To distinguish hyperplastic growth from hypertrophic growth, reliable methods must be used to quantify cell numbers and sizes. For skeletal muscle, the total myofiber number can be directly counted in small, intact neonatal muscles (e.g., *m. soleus*) by cross-sectional scanning of the entire muscle. In large adult muscles (e.g., *m. longissimus dorsi*), direct counting is impossible; therefore, total fiber number is estimated by dividing the total muscle cross-sectional area by the average myofiber area obtained from histology [95].

To overcome the labor-intensive bottleneck of manual histology, recent livestock and meat science studies utilize advanced computer vision and deep learning frameworks for fully automated myofiber segmentation and high-throughput morphometry [96]. Specialized deep learning pipelines, such as Cellpose and the Mask R-CNN-based tool MyoV, leverage convolutional neural networks to perform pixel-level edge detection across large-scale histological images [97]. This technological advancement allows researchers to precisely quantify the total number of myofibers (TNM) across entire muscle landscapes, providing the statistical power needed to confirm true prenatal hyperplastic programming [98].

For intramuscular adipocytes, cell number is mathematically calculated rather than visually counted. This is achieved by dividing the total tissue triglyceride content by the estimated triglyceride content per individual adipocyte [99, 100]. The single-cell lipid content is derived from histologically measured adipocyte diameters, assuming a spherical geometry of lipid-filled cells with a density of 0.9 g/cm^3^ [99, 100]. Together, these histological, computational, and biochemical methods provide a practical framework to evaluate cellular programming in livestock.

## Prenatal programming of muscle fiber architecture

### Molecular and cellular foundations of myogenesis

Skeletal muscle formation during embryonic development begins with signals from the neural tube and notochord [101–103], leading to the commitment of myogenic precursor cells. This process is orchestrated by a hierarchy of transcription factors. Paired-box factors Pax3 and Pax7 mark and regulate muscle progenitor cells, controlling their proliferation and entry into differentiation [104]. The myogenic regulatory factors (MRFs) including Myf5, MyoD, Myogenin, and MRF4, execute the myogenic program, driving the determination, differentiation, and fusion of myocytes into multinucleated myotubes [105–107]. Notably, the expression and activity of these key molecular regulators, particularly the MRFs and upstream signaling pathways (e.g., Wnt/β-catenin), represent critical control points that can be manipulated to alter muscle fiber number during development [108, 109].

Anatomically, muscle fibers are formed in two sequential, prenatal waves. Primary myogenesis generates the initial myotubes that serve as a scaffold [110]. Subsequently, secondary myogenesis occurs, during which new populations of Pax7⁺ fetal myoblasts align and fuse along the primary fibers [110, 111]. This biphasic, stage-specific process is largely completed around birth or hatching in major livestock species, as summarized in Table 1 [112]. After these waves, the formation of entirely new fibers ceases; postnatal growth proceeds primarily through hypertrophy—the fusion of satellite cell-derived myoblasts into existing fibers [113].

**Table 1:** Timing of muscle fiber formation [112]

Species	Primary myofibre formation (gestation/incubation)	Secondary myofibre formation (gestation/incubation)	Full term/Hatching
Pig	Up to ~60 d	~ 54 to 90 d	~ 113 d
Cow	Up to ~125 d	~ 100 d to near term	~ 9 months
Sheep	~ 32 to 38 d	~ 38 to 62 d	~ 5 months
Chicken	From ~6 d	~ 12 to 16 d	21 d
Rainbow trout	Not applicable—muscle fibres do not form in the same biphasic manner. Instead, they exhibit continuous (indeterminate) growth and new fibre production throughout life		

The strict temporal regulation of myogenesis underscores that the window for fundamentally altering muscle fiber number is confined to the prenatal and early neonatal periods. Once the number of fibers is set, postnatal interventions can only modulate fiber size. Therefore, strategies aimed at increasing muscle mass while maintaining meat quality must target these early developmental stages to promote hyperplasia, rather than relying solely on postnatal hypertrophy.

### Prenatal nutritional and genetic strategies to enhance muscle fiber number

In domestic animal production, both the size and number of animal muscle fiber size is increasing, as a result of genetic selection and improved feeding strategy for faster growth in livestock [74, 113]. For example, high-performing piglets have both a greater number and larger size of skeletal muscle fibers than their lower-performing counterparts [114]. As discussed above, excessive muscle hypertrophy is associated with low quality meat and impaired animal health. In addition, postnatal muscle fiber hypertrophy is generally inversely correlated with the number of muscle fibers which are formed prenatally [113]. To stay healthy and high quality, it is critical to elevate muscle growth by muscle hyperplasia rather than solely hypertrophy. Nutritional strategies and genetic selection can change muscle fiber number during the prenatal stages.

#### Prenatal nutrition

Maternal nutrition plays a critical role in determine muscle fiber number [115]. Maternal undernutrition in ewe before muscle fiber formation (d 30 through 70) decreases secondary muscle fiber number of lambs [17, 116, 117]. Maternal undernutrition in late gestation reduce muscle weight but have no effects on muscle fiber number [116]. Increase maternal nutrition supply to pregnant sow increases offspring muscle fiber number and d 25 to d 50 of pregnancy immediately before muscle fiber hyperplasia is the most effective period [115]. However, maternal overnutrition in sow from d 45 to d 85 of gestation (period of secondary muscle fiber formation) leads to a smaller number of type IIB muscle fibers with greater cross-sectional area in offspring pigs [118], and this is likely associated with insulin signaling abnormality [119]. In sheep, maternal overnutrition reduces fetal muscle mass and plasma IGF-1 concentration [120].

IGF-1 promotes myoblast proliferation and skeletal muscle growth of embryonic chickens via the PI3K/Akt signaling pathway [121]. In duck, in ovo IGF-1 injection increases muscle weight, muscle fiber size and SCs number [122]. However, most studies show IGF-1 promotes muscle fiber hypertrophy especially in preventing muscle loss [123, 124], but its role in muscle hyperplasia remains unestablished. Maternal 25-hydroxyvitamin D_3_ (25 OHD_3_) supplementation during gestation increases muscle fiber number in the LT muscle of offspring pigs [125, 126]. While nutrients like vitamin A [127], vitamin C [128], arginine [129], glutamine [130], and some polyphenols [131] are myogenic promoters, whether these nutrients or chemicals can increase muscle fiber number in vivo has not been explored.

#### Genetic selection

Genetic selection has long prioritized improvements in growth performance, with little consideration given to the underlying cellular traits, such as muscle fiber number, in breeding programs. Nevertheless, the historical impact of selecting rapid growth can be seen in comparisons between selected breeds and their unselected ancestors. Over the long term, this selection has generally led to increases in both muscle fiber number and size in species like pigs and chickens [113, 132]. For instance, the European domestic pig exhibits more and larger muscle fibers than its ancestor, the European wild pig [113]. Similarly, broilers possess more numerous and larger muscle bundles than layer chickens [133]. In cattle, Charolais bulls with a high growth index were found to have a greater total number of muscle fibers than those with a lower index [134]. These examples show that the initial, long-term selection for growth rate did, as a secondary effect, increase muscle fiber number. However, this trend has plateaued within modern, intensively selected breeds.

For example, while the meat breed German Angus has larger muscle fibers than the dairy breed Holstein Friesian, there is no significant difference in fiber number between them [74]. More directly, comparisons among modern meat-type pig breeds reveal no significant differences in fiber number [113], This indicates that selection based solely on growth performance has a limited ability to further increase muscle fiber number once a baseline has been established. To substantially increase muscle fiber number beyond current levels and thereby improve growth potential and meat quality, this trait must be explicitly targeted in future breeding indices [113].

DM animals strongly prove that muscle fiber number can be genetically programmed. The discovery of naturally occurring myostatin mutations in Belgian Blue cattle [135], followed by successful gene editing in pigs [136], sheep [137], and chickens [138], demonstrated that a single gene can shift the entire developmental trajectory of myogenesis. The phenotypic outcomes, however, vary with developmental timing. In young animals, increased fiber number dilutes the need for rapid postnatal hypertrophy, transiently reducing fiber size [139, 140]. But as growth proceeds, the same myostatin-deficient myoblasts continue to fuse at an accelerated rate, eventually producing fibers as large as or larger than those of wild-type animals [139, 140]. The early benefit is eroded by sustained lack of braking. MSTN could be an optimal target for programming muscle number and size, if the turning on and off of the gene can be precisely controlled. However, by now MSTN is permanently mutated in all livestock models. The MSTN example underscores both the immense potential and the inherent risks of permanently altering a key regulatory gene early in development. It highlights the need for future strategies that allow for temporally precise and spatially controlled genetic or epigenetic modulation, enabling the benefits of enhanced hyperplasia without the accompanying health and quality trade-offs.

## Neonatal programming of intramuscular adipocyte number

Animal fat is edible and a traditional part of many cuisines, but its consumption is shifting from a necessity for energy in the past to a culinary choice for flavoring. Fat deposition is closely correlated to animal health, meat quality and the feed efficiency [141]. As lean tissue gain is four times as efficient as the accretion of fat tissue, the deposition of fat reduces feed efficiency [2]. To the animal itself, excessive fat deposition causes fatty liver, which interrupts whole-body energy homeostasis and negatively affects meat quality in terms of water holding capacity and meat tenderness [142]. To human beings, although the risk has not been rigorously verified, people are suggested to intake less fat in concern of obesity related health problems [143]. Although the acceptability varies in individuals, visceral and subcutaneous fat is often inedible and is less desirable in livestock production. However, IMF is popular because it improves meat juiciness and flavor, and enhanced sensory quality, like mouthfeel and taste [144], meeting consumer preferences [145]. To produce marbled meat, producers feed animals a high-energy diet at the finishing stage. However, this approach inevitably increases the overall body fat mass, leading to a decrease in meat yield [13, 14]. Strategies that increase IMF without increasing fat in other depots are needed.

The number and size of fat cells are also crucial for meat quality, particularly marbling, which contributes to flavor and juiciness [146]. A larger number of smaller fat cells results in finer, more even marbling throughout the muscle, which is often associated with higher-quality steaks [146]. Steaks with "fine" marbling have significantly smaller adipocytes than those with "coarse" marbling [146]. The even distribution of smaller fat cells provides a more intricate and desirable fat distribution [146].

### The biology of adipose tissue: development, expansion, and depot specificity

Adipogenesis is the process by which mesenchymal stem cells (MSCs) commit to the adipocyte lineage and terminally differentiate into mature adipocytes. This process is orchestrated by a tightly regulated cascade of transcriptional events. Bone morphogenetic proteins (BMPs) initiate preadipocyte commitment by guiding MSCs toward distinct adipose lineages: BMP2 and BMP4 promote a white adipocyte fate [147], while BMP7 directs MSCs toward a brown adipocyte lineage [148, 149]. The transcription factor ZFP423 plays a critical role in committing MSCs to the adipocyte lineage [150]. In undifferentiated MSCs, ZFP423 is sequestered in the cytoplasm through its interaction with WISP2 (WNT1 inducible signaling pathway protein 2) [151]. BMP signaling, via BMP2 or BMP4, activates receptor-mediated phosphorylation of SMAD1/5/8. The phosphorylated SMAD complex then binds to ZFP423, displacing WISP2 and allowing ZFP423 to translocate into the nucleus, where it promotes PPARγ expression and lineage commitment [147, 150, 151].

Terminal adipogenesis is well-characterized in models such as the 3T3-L1 cell line [152]. Induction with insulin, dexamethasone, and IBMX rapidly upregulates C/EBPβ and C/EBPδ, which in turn induce the expression of *PPARγ* and *C/EBPα* [153–155]. As the master regulator of adipogenesis, PPARγ is both necessary and sufficient for this process [154]. PPARγ works in a synergistic positive feedback loop with C/EBPα to ensure the full expression of the mature adipocyte phenotype [156] by activating downstream target genes involved in lipid uptake and storage, such as FABP4, adipsin, CD36, and lipoprotein lipase [157–159].

Unlike muscle fiber numbers, which become fixed shortly after birth, the number of fat cells is not "set". Adipose progenitors continue to proliferate, meaning that fat can continue to grow via both hyperplasia and hypertrophy throughout life [160]. However, the proliferation rate of adipose progenitors, especially in visceral and subcutaneous fat, slows significantly after birth [20]. During the finishing phase, when animals are fed high-energy diets, the dramatic increase in fat mass is primarily due to the hypertrophy of existing fat cells [160]. In humans, adipocyte numbers are set during childhood and are tightly regulated in adults [161]. Although approximately 10% of fat cells are renewed annually, the total number of fat cells stays constant in adults [161]. In cattle, a significant increase in cell number was observed from 15% to 45% maturity, especially in subcutaneous fat [162]. However, adipose cells became 15 times greater from 15% to 65% mature weight, whereas total adipose cell number increased only 1.8-fold, indicating that the growth of adipose tissue at this stage mainly relies on adipocyte hypertrophy [162].

The timeline of adipose tissue development in different anatomical positions varies. Although the time point has not been accurately determined, it is widely accepted that most subcutaneous and visceral adipocytes are formed before birth in ruminant animals [18, 20, 163, 164], while the intramuscular adipocytes are formed after birth [20]. In beef cattle, perirenal fat is detectable as early as 80 days of gestation [165]. Intramuscular adipocytes are the last to form and accumulate lipids, and intramuscular adipogenesis in beef cattle is estimated to occur around 180 d of gestation until about 250 days of age [18, 165]. Direct quantification of adipocyte number remains technically challenging. Therefore, studies typically estimate it indirectly: by measuring fat tissue weight and calculating adipocytes per gram, based on average cell volume and tissue density [166]. Using this method, researchers observed that hyperplasia in subcutaneous and perirenal adipose tissue of cattle is complete by about 8 months of life, but is still active in interfascicular adipose tissue at 14 months of life [166].

### Determinants of fat deposition: genetics, sex, and nutrition

Fat deposition is affected by many factors, including genetics, sex, management, and nutritional factors. Pigs are characterized by a high proportion of subcutaneous fat, which constitutes approximately 70% of their total body fat. In contrast, sheep have a more balanced final fat distribution, with internal depots (kidney, omental, and mesenteric) making up a combined 33%, which is a significantly larger share than in pigs. In cattle, beef breeds like Hereford deposit more subcutaneous fat, while dairy breeds such as the Jersey deposit a much higher proportion internally. For all three species, intramuscular fat (marbling) represents a minor and late-maturing depot [167]. Japanese black cattle contain over 20% IMF in the LD muscle, while other breeds including Angus, Holstein–Friesian, and Belgian Blue contain less than 5% [168].

Females generally deposit more total fat than males, especially in beef cattle, sheep, and poultry, though specifics vary by species and castration status [8]. Heifers (females) often have higher IMF than bulls (intact males), while steers (castrated males) show intermediate levels [169, 170]. To maximize marbling, producers practically feed steers a high-energy diet for high-quality meat production [171]. Heifers are also excellent choices for marbling meat production [171]. Ewes also have more fat than rams [172] and require more trimming of subcutaneous fat after slaughter [173]. The difference in backfat depths between male and female piglets is minor; however, males had higher IMF and liver fat than females [174]. In addition, castrated piglets showed higher backfat thickness and intramuscular fat content [175], exhibiting a different pattern compared to cattle and sheep.

Animal maturity is a critical factor affecting fat deposition. As animals mature, fat deposition generally increases significantly, especially after muscle and bone growth slows down, with energy surplus being stored as fat [8, 176, 177]. The percentage of body fat is highly correlated with body weight. Specifically, the total fat of cattle increases by 0.05% while that of sheep increases by 0.52% with every kilogram increase in body weight [177]. In sheep, the amount of intramuscular fat in the *longissimus thoracis* muscle significantly increased as the sheep became older, reaching 5.5% at 22 months of age, comparing to 3.93% at 4 months of age [178]. However, the proportion of IMF in relative to total carcass fat decreases as animals mature, indicating a higher deposit rate in other fat depots [178].

Animal nutrition is no doubt the primary factor that determines fat accretion level. Besides the energy provided by the feedstuff, many active energy regulatory nutrients like fatty acids, vitamins, and amino acids play important roles in fat accumulation of livestock animals. Vitamin D is a well-known inhibitor of adipogenesis, vitamin D deficiency is associated with excessive fat accumulation [179]. The metabolite of vitamin D, 1,25-dihydroxyvitamin D, activates its nuclear receptor, vitamin D receptor, to block adipogenesis by down-regulating C/EBPβ expression [180–182]. Maternal vitamin D supplementation to sows (from 41 d to birth) reduces backfat thickness of offspring but increased IMF content [183]. Fatty acids, especially polyunsaturated fatty acids (PUFAs) and their oxidized derivatives, promote adipogenesis by acting as natural ligands for PPARγ [184]. For example, conjugated linoleic acid (CLA) increased both intramuscular and subcutaneous fat content in finishing pigs [185]. Protein restriction increases fat accumulation by inhibiting muscle growth. As early as 1994, Castell et al. [186] found that feeding low-protein diets during growth and finishing phases increased intramuscular fat content. Subsequent studies confirmed that low-lysine [187, 188] or low-protein diets can elevate intramuscular fat in pigs.

While these factors collectively influence the final pattern of fat deposition, most exert their primary effects during the phase of adipocyte hypertrophy (lipid filling). In contrast, targeting the earlier, determinative phase of adipocyte hyperplasia, particularly during the neonatal window for IMF, through focused nutritional programming (e.g., vitamin A supplementation) represents a more fundamental and depot-specific strategy to enhance marbling without increasing overall adiposity.

### Fine marbling without excess overall fat deposition

While a small amount and evenly distributed fat tissue between and inside muscle tissue provides favorable flavor, large pieces of fat tissue are unwanted and sometimes inedible to most people. For pork and lamb, the cuts are relatively smaller. Fats such as the subcutaneous and intermuscular fat are often retained and cooked together with the lean meat, as they are integral to cuts like pork belly or lamb shoulder, which are valued for their interlayering of fat and muscle. In contrast, beef primal cuts are much larger. The subcutaneous and intermuscular fat are typically trimmed away during butchering, making them less valuable. Therefore, for beef, it is primarily the intramuscular fat that is evenly distributed between the muscle fibers, that is crucial for enhancing tenderness, juiciness, and flavor. For most livestock animals including cattle, sheep, pig and poultry, visceral fat is undesirable.

Fattening is practical to increase the weight and improve the meat quality of livestock animals, especially sheep and cattle. A high-energy diet feeding during the fattening stage improves meat quality by increasing IMF accumulation but often leads to excessive accumulation of visceral and subcutaneous fat. Increasing IMF without overall fat accretion is not easy. Genetically, there is a positive genetic correlation between subcutaneous fat thickness and IMF in sheep [189]. Only in wagyu cattle, there is a weak negative correlation between subcutaneous fat thickness and IMF score [190], but the underlying mechanism is unclear. There are few nutrients that appear to differentially regulate adipogenesis of cells isolated from different fat depots. For example, CLA promotes adipogenic gene expression in porcine intramuscular stromal vascular fraction (SVF) cells while suppressing it in subcutaneous SVF cells [191], but this has not been validated in animal trials. Early in vitro experiments found that compared to acetate (a rumen fermentation product), intramuscular adipose tissue in beef cattle preferentially uses glucose (a product of grain digestion) for fat synthesis [192]. Nonetheless, substituting a high-energy feed for an equivalent amount of energy from pasture did not elevate IMF in beef cattle [193]. Numerous evidence suggests that fat depots expand at a similar rate during the fattening stage [193–195]. To date, no effective nutritional strategy has been identified to target lipid deposition specifically in intramuscular adipocytes.

**Fig. 3:**
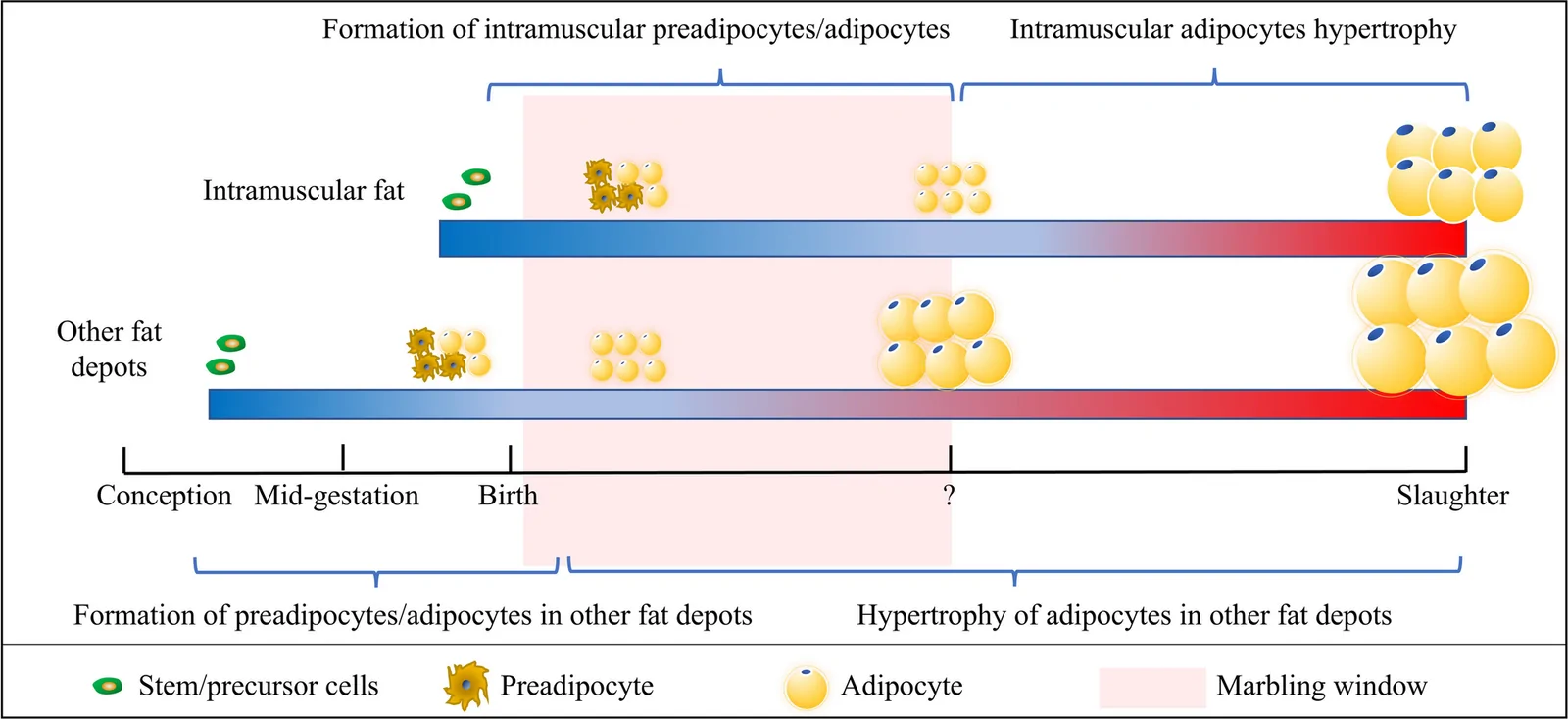
The timing of adipocyte formation and lipid accumulation in different fat depots

In most livestock studies, more attention has been paid to lipid accumulation (hypertrophy) during the fattening stay while the process of adipocyte formation (hyperplasia) has been ignored. As we previously mentioned, the timing of adipogenesis varies at different depots. Subcutaneous and visceral adipocytes are mostly formed prenatally [18, 20] while intramuscular preadipocytes start to proliferate and differentiate into adipocytes around birth until late life [18, 20, 196]. This developmental timing difference offers a promising strategy to selectively enhance IMF deposition by increasing intramuscular adipocyte number [163]. As empirical evidence for this theory, neonatal vitamin A administration between birth and one month of age increased intramuscular adipocytes and enhanced intramuscular fat accumulation by 45% and 82% in cattle [100, 197, 198] and sheep [100], respectively, without elevating subcutaneous and visceral fat accumulation. Of note, this has been verified by other independent studies performed in Korea [199] and Brazil [200, 201] using different cattle breeds. Figure 3 summarizes the timing of adipocyte formation and lipid accumulation in different fat depots.

Thus, the ideal fat distribution should aim to moderately increase and evenly disperse intramuscular fat while suppressing excessive accumulation in subcutaneous and visceral depots. This underscores the necessity of viewing muscle and adipose development as an integrated system, where coordinated manipulation of both cell number and size is key to achieving efficient, high-quality, and healthy meat production.

### Nutritional programming of intramuscular adipocytes

The concept of nutritionally programming adipocyte number finds its strongest validation in neonatal vitamin A studies. Unlike nutrients that broadly affect all fat depots, vitamin A administered during the neonatal window exemplifies depot-specific, time-sensitive programming, which precisely expands the intramuscular adipocyte pool without increasing subcutaneous or visceral fat [100, 197, 198]. This makes it a cornerstone example of the DCP approach.

An expanded intramuscular preadipocyte and adipocyte pool provides more storge units for lipid accumulation during the fattening stage. The potential of intramuscular fat accumulation of the livestock can be nutritionally manipulated at the neonatal stage when this potential is hard to be evaluated in practical. Adipose tissue is highly vascularized [202] and the surrounding capillaries not only supply nutrients, oxygen, and hormones while removing waste [20, 203], and also act as a stem cell/progenitor pool for adipocyte replenishment and expansion in adipose tissue [204, 205]. Pericytes and endothelial origin cells were found to differentiate into adipocytes [206, 207]. The adipose progenitors marked by PDGFRα^+^ [208], PDGFRβ^+^ [209], NG2 [210] are all located surrounding the blood vessels. Effective vascular development is required for adipose tissue development [211] and is considered as the rate-limiting step in adipocyte formation [212].

In beef cattle, vascular density increases significantly with marbling score [213]. Wagyu cattle, the benchmarked marbling breed, exhibit greater capillary abundance than leaner breeds [214]. In both cattle and sheep, neonatal vitamin A injection significantly promotes angiogenesis in skeletal muscle and increased the density of PDGFRα^+^ preadipocytes [100, 197]. Histologically, marbling flecks locate proximity to blood vessels [94], an increased blood vessel density would not only increase intramuscular fat content but may also contribute to fine marbling distribution.

Our recent knowledge about nutrients on adipogenesis is majorly generated from in vitro studies mostly performed using 2D cell cultures, which lack the multicellular interactions present in vivo. We previously developed a vascularized adipose organoid model using sheep SVF cells to simulate IMF development in vivo [215]. Using this model, we investigated the regulatory effects of dietary nutrients on sheep IMF deposition and identified VC, VE, VK1, GAA, Leu, Lys, Met, NCG, Trp, ALA, LA, c9, t11-CLA, HAc, NaAc as pro-angiogenic nutrients which enhance preadipocyte formation by promoting vascular development, VB9, VD, VK2, Tau, and NaBu as anti-angiogenic nutrients. In addition, VC, VE, VK1, VK2, GAA, Leu, NCG, Trp, ALA, LA, and HAc promotes adipocyte differentiation, with VE, VK1, GAA, Leu, LA, and HAc further enhancing lipid accumulation [216].

These findings suggest a stage-specific nutritional programming framework: pro-angiogenic and pro-adipogenic nutrients can be strategically supplemented during the neonatal window to establish intramuscular adipogenic capacity, while pro-lipogenic nutrients can be reserved for the finishing phase to maximize lipid filling. Conversely, dietary anti-angiogenic, anti-adipogenic, and anti-lipogenic nutrients should be minimized during their respective sensitive periods. However, translating these cell-centric findings to whole-animal production necessitates rigorous in vivo validation within the context of systemic growth, metabolism, and livestock welfare. To bridge this operational gap, livestock validation trials are currently underway within our laboratory to systematically evaluate the physiological efficacy of these organoid-selected nutrients. Furthermore, a collaborative effort from the wider scientific community is strongly encouraged to functionally validate these candidate molecules across diverse breeds and farming systems, thereby collectively accelerating the development of precision nutritional strategies for early-life cellular programming.

## Integrated developmental programming: coordinating muscle and adipose development

Skeletal muscle fibers and intramuscular adipocytes are the fundamental building blocks of meat. Their ratio, size, and spatial arrangement within the tissue collectively determine the sensory and nutritional quality of the final product [144, 146]. The preceding chapters have established that increasing the number of muscle fibers and intramuscular adipocytes, controlling their hypertrophic expansion, ensuring even distribution of marbling flecks, and supporting the development of vascular networks are essential for architecting a piece of meat that is both tender and flavorful [94, 146, 197].

In commercial production, however, muscle growth and fat deposition are often positioned as opposing forces. Genetic selection for rapid lean accretion suppresses intramuscular adipogenesis [8, 217]; intensive finishing for marbling increases subcutaneous and visceral fat, reducing lean meat yield [13, 14]. This antagonism has long been accepted as an inevitable trade off, and postnatal nutritional or genetic interventions have struggled to escape it [193, 195]. However, a critical observation reframes the problem: the negative correlation between muscle and fat manifests primarily during postnatal growth. Mesenchymal stem cells and their progeny contribute to both myogenic and adipogenic lineages during development [218, 219], but these contributions are temporally segregated: myogenesis dominates prenatally, while intramuscular adipogenesis extends into postnatal life [18, 20, 163]. This temporal separation, coupled with a shared progenitor origin, makes the fetal and neonatal periods opportune windows for programming both muscle and marbling potential through appropriately timed interventions.

Our recent work provides direct evidence for this possibility. Neonatal vitamin A administration, by enhancing angiogenesis and expanding progenitor pools, promotes both skeletal muscle growth and intramuscular fat deposition, without increasing subcutaneous or visceral fat [100, 127, 197, 198]. This demonstrates that cooperative programming of muscle and adipose lineages is not only theoretically plausible but practically achievable. The DCP framework therefore offers a pathway beyond the postnatal trade off. By targeting critical windows of cell differentiation, when stem cells commit to myogenic or adipogenic fates, it becomes possible to architect meat at the cellular level, establishing a tissue foundation that supports both high yield and superior quality before the competitive logic of postnatal growth takes hold.

### The competitive logic of muscle–adipose interaction

In livestock, a high speed of muscle growth is generally negatively correlated with fat accumulation, particularly IMF [8, 217], a relationship further evidenced by the negative correlation between the cross-sectional areas of both the whole muscle [220] and individual muscle fibers [221] with IMF content. These interactions are mediated through direct cellular communication and secretory factors. In vitro, mature adipocytes inhibit myogenesis [222], while muscle conditional medium inhibits adipogenesis and lipid accumulation [223]; specifically, co-culture with satellite cell-derived myofibers strongly inhibits adipogenesis of intramuscular PDGFRα^+^cells [218]. This crosstalk is further regulated by myokines, including interleukin-15 [224], myostatin [225], and irisin [226], which inhibit adipogenesis and lipid accumulation. In addition, IL-15 stimulates the proliferation of fibro-adipogenic progenitors (FAPs) [227] while myostatin promotes fibrogenesis [228]. In a supportive feedback loop, FAPs release FGF7 to promote satellite cell proliferation [229]. FAPs are required for the maintenance of both skeletal muscle and the MuSC pool [230]. A central challenge is whether both increased muscle growth and enhanced intramuscular fat deposition, two traits which are often negatively correlated, can be achieved simultaneously to improve overall meat quantity and quality.

### Vascularization: a shared control point

The synchronized coordination between myogenesis and adipogenesis implies the existence of common physiological mediators that transcend individual tissue boundaries to control early-life developmental programming. Among these shared control points, vascularization serves as an active, instructive signaling hub rather than a merely passive nutrient delivery system to systematically regulate the parallel expansion of muscle and adipose tissues. Histologically, capillary density is positively correlated with both satellite cell abundance and PDGFRα⁺ preadipocyte density, as endothelial cells establish a shared physical and paracrine scaffold that directly dictates both myofiber growth and lipid deposition [43, 231, 232]. Within these specialized microenvironments, endothelial cells and pericytes provide vital niche signals that maintain progenitor quiescence, support self-renewal, and regulate lineage commitment [40, 204, 205]. In muscle tissue, juxtavascular satellite cells exhibit a significantly higher regenerative capacity [42], whereas in adipose tissue, pericytes and endothelial-derived cells contribute directly to the resident adipocyte progenitor pool [206, 207, 209].

While these anatomical associations provide strong spatial clues, robust in vivo genetic and pharmacological models have established a direct causal link between active angiogenesis and precursor cell proliferation. Specifically, the expansion of fibro-adipogenic progenitors and preadipocytes is tightly gated by endothelial-derived angiocrine factors, including platelet-derived growth factor BB (PDGF-BB), insulin-like growth factor 1 (IGF-1), and basic fibroblast growth factor (bFGF) [40, 233]. Functional in vivo studies have demonstrated that the targeted deletion of endothelial VEGF-A or the pharmacological blockade of vascular endothelial growth factor receptor 2 (VEGFR2) signaling not only arrests capillary sprouting but simultaneously severely impairs the mitotic clonal expansion of adjacent muscle stem cells and preadipocytes, leading to a marked ablation of both stem cell niches and adipose tissue formation [234, 235].

This underlying causal mechanism provides a profound theoretical foundation for targeted nutritional interventions in livestock production. For instance, neonatal vitamin A supplementation enhances intramuscular adipogenesis by promoting angiogenesis and expanding the preadipocyte pool [100, 197–201], while simultaneously stimulating muscle growth by increasing satellite cell numbers and enhancing their myogenic activity [127, 199, 236]. This dual effect is mediated through a dramatic improvement in capillarization [197], which correlates directly with the abundance of both satellite cells [231] and adipose progenitors [232]. Besides vitamin A, recent in vitro evidence has identified a wide array of pro-angiogenic nutrients, including vitamin C, vitamin E, vitamin K1, guanidinoacetic acid, leucine, lysine, methionine, N-carbamoylglutamate, tryptophan, alpha-linolenic acid, linoleic acid, *cis*-9,*trans*-11 conjugated linoleic acid, acetic acid, and sodium acetate, which actively promote the capillarization of stromal vascular fraction cells derived from sheep muscle [216]. These nutrients could potentially contribute to the establishment of the muscle and adipose stem/precursor cell pools and enhance the formation of both cell types. Consequently, while muscle and fat deposition traditionally exhibit a competitive relationship in livestock development, strategically promoting angiogenesis via precise early-life nutrition creates a synergistic effect that drives the simultaneous formation of both muscle and fat cells, successfully unifying these two growth pathways within the framework of coordinated developmental programming.

### Stage-specific programming of muscle and adipose cellularity

**Fig. 4:**
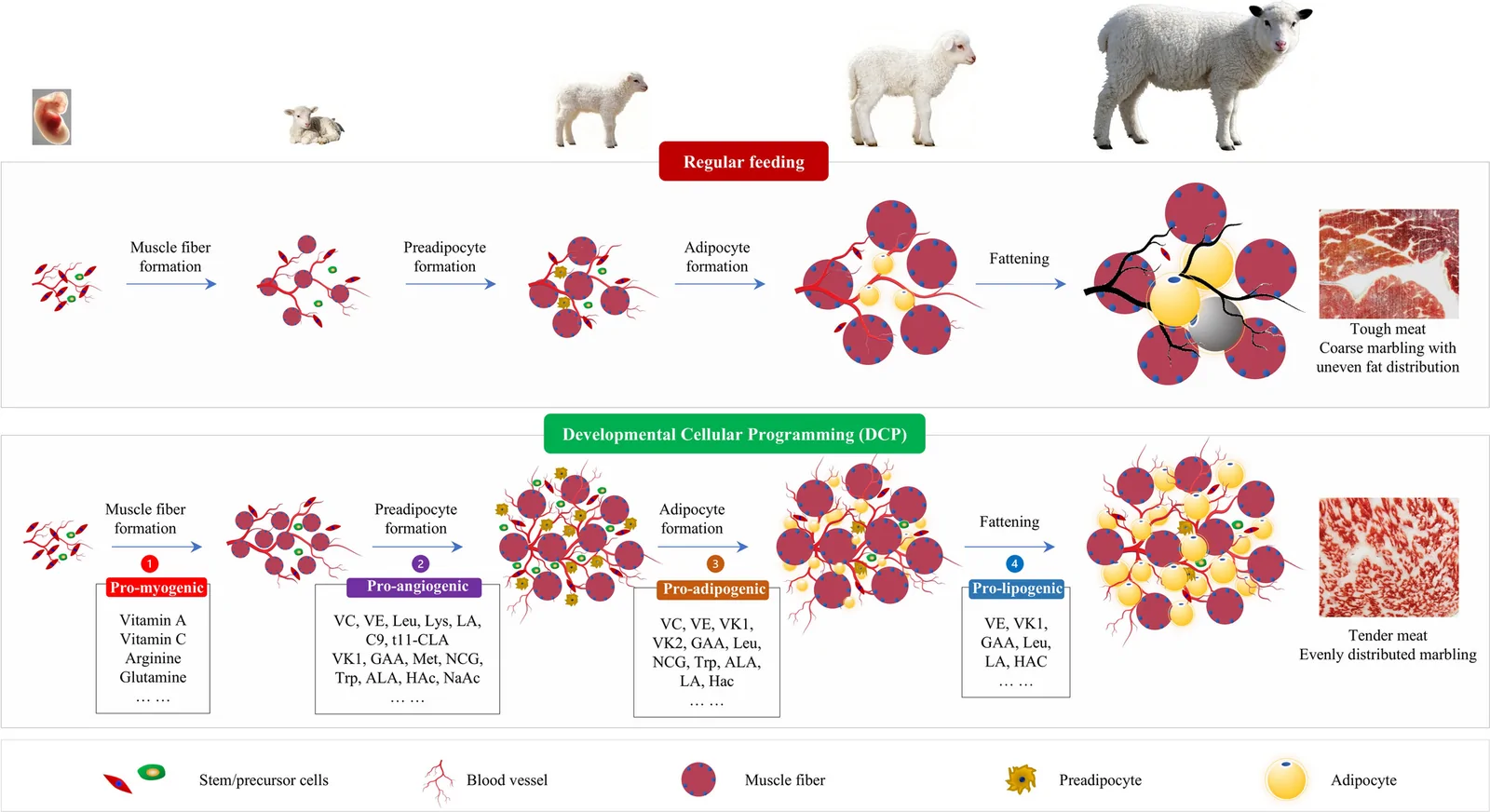
The Developmental Cellular Programming (DCP) framework: stage-specific nutritional programming of muscle fiber number and intramuscular adipogenic potential

Skeletal muscle cells and adipocytes are formed at different stages, precise nutrition programs the development of muscle and fat that contribute to meat production [164]. Figure 4 illustrates the DCP framework in practice, showing how pro-myogenic nutrients at embryonic/fetal stages program muscle fiber number, while pro-angiogenic/adipogenic nutrients at the neonatal stage program intramuscular adipocyte potential. As discussed above, increasing the number of muscle fibers and intramuscular adipocytes, while limiting the formation and energy deposition of visceral and subcutaneous fat meet the industrial demand for high production, high quality and efficiency. The embryonic and fetal stages are the time for programming muscle fiber number while the neonatal stage is the optimal time to improve intramuscular adipogenic potential. The pro-myogenic nutrients provided at the embryonic and fetal stages could potentially enhance muscle growth potential by increasing muscle fiber number, and pro-angiogenic and adipogenic nutrients provided at the neonatal stage could increase intramuscular adipocytes without affecting other fat depots. At the fattening stage, the anti-lipogenic nutrients should be restricted for ensuring fat accumulation. This is why vitamin A is supplemented to cattle and sheep at the neonatal stage [100, 197, 198, 200, 236] while a vitamin A deficient diet is provided to finishing beef cattle to enhance intramuscular lipid accumulation [237–241]. Importantly, this DCP philosophy is globally applicable across diverse production models, ranging from intensive barn-feeding to extensive grazing systems. In pastoral regions, grazing livestock frequently encounter severe nutritional restrictions and vitamin deficiencies during the prolonged winter cold season, which directly limits offspring myofiber hyperplasia and marbling cellularity development. Rather than continuous total dietary regulation, implementing targeted, strategic supplementation during these narrow critical windows provides a highly feasible and economic approach to rescue and program lifetime performance, effectively overcoming the severe nutritional suppression caused by winter forage scarcity.

By programming the muscle and intramuscular cell number and size, the ultimate meat is composed of evenly distributed intramuscular adipocytes between skeletal muscle fibers in small sizes. Of note, within this stage-specific programming framework, the expansion of precursor cellularity operates within an integrated multicellular niche rather than isolated lineage propagation. Here, active angiogenesis serves as a central coordinating axis that functions in close spatiotemporal tandem with other supportive or regulatory cell types, including resident macrophages and fibro-adipogenic progenitors, to gate progenitor commitment. Through the coordinated secretion of angiocrine factors and local regulatory factors, this cellular interactome systematically orchestrates extracellular matrix remodeling and growth factor bioavailability, thereby defining the absolute structural and lifetime potential of both myofiber and adipocyte pools.

## Conclusion

In summary, advancing livestock production requires the DCP paradigm, which prioritizes active architectural programming over passive postnatal regulation. Because the critical developmental windows for establishing myofiber and adipocyte numbers are predominantly prenatal and neonatal, they establish a cellular blueprint that postnatal interventions cannot fully reshape. Therefore, future efforts must focus on strategically programming myogenesis and adipogenesis during these early stages through nutritional interventions. For instance, maternal and neonatal vitamin A supplementation has demonstrated potential to enhance intramuscular adipocyte number and angiogenesis, thereby improving marbling without elevating subcutaneous or visceral fat. The interplay between muscle and fat, mediated by vascular-stromal networks and multi-cellular secretory factors, positions angiogenesis as a shared control point that synergistically supports both muscle growth and intramuscular fat deposition within the developmental niche. Moving forward, validating and refining DCP protocols across species through the integration of advanced multicellular vascularized organoids, biomimetic models, and live-animal trials will be essential. By precisely designing cellular architecture at its developmental origins, this programming-based approach provides a robust framework to simultaneously optimize animal health, production efficiency, and meat quality.

## Data Availability

No datasets were generated or analysed during the current study.

## Abbreviations

Akt: Protein kinase B
BBDM: Belgian Blue double-muscled
BMP: Bone morphogenetic protein
C/EBP: CCAAT/enhancer-binding protein
CLA: Conjugated linoleic acid
DCP: Developmental Cellular Programming
DM: Double-muscled
ECM: Extracellular matrix
FABP4: Fatty acid-binding protein 4
FAPs: Fibro-adipogenic progenitors
FCR: Feed conversion ratio
FGF7: Fibroblast growth factor 7
GAA: Guanidinoacetic acid
HAc: Acetic acid
IBMX: 3-Isobutyl-1-methylxanthine
IGF-1: Insulin-like growth factor 1
IL-6: Interleukin-6
IMF: Intramuscular fat
K^+^: Potassium ion
LA: Linoleic acid
Met: Methionine
MSCs: Mesenchymal stem cells
MSTN: Myostatin
MRFs: Myogenic regulatory factors
Na^+^/K^+^: Sodium-potassium pump
NaAc: Sodium acetate
NaBu: Sodium butyrate
NCG: N-carbamylglutamate
NG2: Neuron-glial antigen 2
PAX: Paired box
PDGFR: Platelet-derived growth factor receptor
PI3K: Phosphoinositide 3-kinase
PKC: Protein kinase C
PPARγ: Peroxisome proliferator-activated receptor gamma
PUFAs: Polyunsaturated fatty acids
SCs: Satellite cells
Shh: Sonic hedgehog
SVF: Stromal vascular fraction
TNF-α: Tumor necrosis factor alpha
Trp: Tryptophan
VC: Vitamin C
VE: Vitamin E
VK1: Vitamin K_1_
VK2: Vitamin K_2_
WB: Wooden breast
WISP2: WNT1 inducible signaling pathway protein 2
WS: White striping
Wnt: Wingless-related integration site
ZFP423: Zinc finger protein 423
